# Nutrition and Supplement Update for the Endurance Athlete: Review and Recommendations

**DOI:** 10.3390/nu11061289

**Published:** 2019-06-07

**Authors:** Kenneth Vitale, Andrew Getzin

**Affiliations:** 1 Department of Orthopaedic Surgery, Division of Sports Medicine, University of California San Diego, La Jolla, CA 92037, USA; 2 Sports Medicine, Cayuga Medical Center, Ithaca, NY 14850, USA; agetzin@cayugamed.org

**Keywords:** athletes, physical endurance, sports nutritional sciences, nutritional requirements, dietary supplements

## Abstract

Background: Endurance events have experienced a significant increase in growth in the new millennium and are popular activities for participation globally. Sports nutrition recommendations for endurance exercise however remains a complex issue with often opposing views and advice by various health care professionals. Methods: A PubMed/Medline search on the topics of endurance, athletes, nutrition, and performance was undertaken and a review performed summarizing the current evidence concerning macronutrients, hydration, and supplements as it pertains to endurance athletes. Results: Carbohydrate and hydration recommendations have not drastically changed in years, while protein and fat intake have been traditionally underemphasized in endurance athletes. Several supplements are commercially available to athletes, of which, few may be of benefit for endurance activities, including nitrates, antioxidants, caffeine, and probiotics, and are reviewed here. The topic of “train low,” training in a low carbohydrate state is also discussed, and the post-exercise nutritional “recovery window” remains an important point to emphasize to endurance competitors. Conclusions: This review summarizes the key recommendations for macronutrients, hydration, and supplements for endurance athletes, and helps clinicians treating endurance athletes clear up misconceptions in sports nutrition research when counseling the endurance athlete.

## 1. Introduction

Participation in endurance events has increased both nationwide and globally, with 2.5 million triathlon participants in the US in 2015 [1] and 3.5 million individuals worldwide [2]. In recent years, there has been a shift in running from standard marathon races to “other distance” races such as mud runs, color runs, and obstacle course races [3]. Furthermore, ultra-endurance events are also gaining popularity [1,3]. Ultra-endurance activities are typically defined as events lasting at least 4 [4] to 6 h [5] duration. Prior studies have illustrated the challenges that ultra-endurance exercise exerts on the body in terms of fatigue, sub-optimal nutrition, and energy deficit [4,5], and brings awareness to the potential medical complications of ultra-endurance exercise [5] underscoring the importance of an individualized nutritional approach [4]. Due to the popularity of endurance and ultra-endurance events, there is a need to define nutritional needs of the athletes. Our goal with this review is to provide the reader with a comprehensive review in a consolidated form and offer practical, evidence-based recommendations that are valuable and directly applicable to the clinician involved in athlete care.

Although there have been significant advances in the understanding of nutritional requirements for endurance athletes, many gaps still exist in the literature. The science of nutrition remains a complex topic, continually evolving, and sometimes contradictory. “Sports nutrition” involves the fields of sports medicine, sports science, dietetics, cultural influences, and even popular media. Especially when it comes to elite athletes and specifically “what to eat,” nutritionists, registered dietitians, sports scientists, physicians, and other healthcare professionals often debate on the ideal diet.

This review summarizes the current available evidence regarding macronutrients and highlights new areas of research regarding select supplements of interest to endurance athletes. The goal is to bring clarity in areas of uncertainty involving optimal nutrition for endurance exercise, and to provide recommendations for athletes trying to optimize their health and performance. To cover every vitamin, mineral, and supplement available in the arena of “nutrition for athletes” however is beyond the scope of this paper, so this review shall focus on the major macronutrient requirements including carbohydrate (CHO), protein and fat needs, hydration requirements, and select specific topics including caffeine, nitrates, probiotics, and antioxidants as they relate to endurance athletes. In the authors’ clinical experience treating endurance athletes, these topics were included to provide clinicians with information regarding the more commonly asked questions by endurance athletes. In reality there are numerous supplements and strategies that may be employed by endurance athletes. However, in the authors’ experience caffeine and nitrates are frequently investigated and used by athletes. Furthermore, antioxidants and probiotics are commonly explored topics by injured athletes and are steadily-growing fields of research. Lastly, “hot topics” and controversial views in endurance exercise are covered such “train low” vs. “train high” states, branched-chain amino acids vs. essential amino acids, vegetable vs. animal vs. milk proteins, and drinking to plan vs. drinking to thirst.

## 2. Materials and Methods

**Figure 1:**
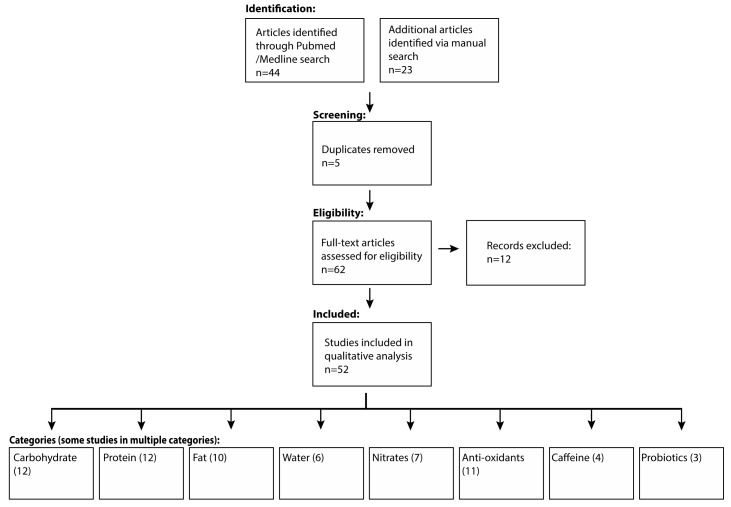
PRISMA flow diagram search strategy.

A PubMed/Medline search was performed for articles between 1980 and December 2018 using search terms “nutrition for athletes,” “sports nutrition,” “endurance athlete nutrition,” “supplementation endurance,” and the above MeSH keywords, without restrictions on language, sex or age. Additionally, references of extracted articles were manually searched. Sixty-seven articles were retrieved, duplicates were removed, and the remaining articles were screened for relevance. Twelve articles were excluded as they either contained clinical studies on non-athletes, or mainly non-endurance (strength and power) athletes or were animal-based studies. Fifty-two were included and further categorized into macronutrients, hydration, and supplements; see Figure 1 for the PRISMA search strategy. A systematic review was not done due to extreme heterogeneity of studies and data; a clinical/descriptive review was thus performed.

From the selected studies, clinical recommendations are provided according to the American Academy of Family Physicians’ Strength-of-Recommendation Taxonomy (SORT) grading scale [6]. The scale levels are derived from the 2002 United States Department of Health and Human Services’ Agency for Healthcare Research and Quality (AHRQ) report that addresses three key research elements: quality, quantity, and consistency of evidence [7]. Most studies were position stands, reviews, and current best-evidence statements of several international organizations (see below) with Level 1 “consistent and good quality patient-oriented evidence” data with a strength of recommendation (SORT) rating of A.

Sections are divided into Carbohydrate (with subsections on pre-competition “loading” vs. during competition “fueling” requirements), Protein (subsections on daily vs. pre-, during, and post-exercise requirements), Fat, Hydration, and finally Supplements and “Hot Topics.” When available, new and controversial or opposing views to traditional sports nutrition recommendations are presented. This better provides the clinician with up-to-date knowledge of progressive and alternative options that endurance athletes may seek in attempt to improve health and athletic performance.

## 3. Results

### 3.1. Carbohydrate

Carbohydrate requirements for the endurance athlete can be a fiery topic, often leading to passionate (and sometimes confrontational) debates on ideal intake amongst the fitness and medical community. The joint position stand of the Academy of Nutrition and Dietetics (AND), Dietitians of Canada (DC), and the American College of Sports Medicine (ACSM) recommends that moderate exercise (1 h/day (h/day)) requires 5–7 g per kilogram of bodyweight per day (g/kg/day) of CHO, while moderate to high intensity exercise (1–3 h/day) mandates 6–10 g/kg/day. Ultra-endurance athletes with extreme levels of commitment to daily activity (4–5 h of moderate to high intensity exercise every day) may need up to 8–12 g/kg/day [8]. The International Society of Sports Nutrition (ISSN) recommends in order to maximize glycogen stores athletes should employ an 8–12 g/kg/day high CHO diet [9].

Carbohydrate (as blood glucose and muscle glycogen) has the advantage of generating more ATP per volume of oxygen (O_2_) compared to fat [10] but exhaustion of liver and muscle CHO stores is associated with fatigue, reduced work, and impaired concentration [8,11,12]. It is the often-described feeling by athletes of “hitting the wall,” or “bonking.” Therefore, fueling strategies both before and during the race/event have been developed and outlined below. An important point to the clinician however, is that even after 4.5 h of cycling at 70% of maximum O_2_ consumption (VO_2max_) when CHO stores should be entirely depleted, elite athletes can still run at 16 km/h for an additional 2.5 h at 66% VO_2max_ [13]. Consequently, glycogen depletion must not be the sole determiner of fatigue. Other CHO sources such as lactate utilization and other mechanisms such as increased capability to oxidize fat (see below) are postulated [14] to account for this effect and clinicians should consider this when counseling athletes.

#### 3.1.1. Pre-Competition, “Loading”

Prior to the race (if the event is to last <90 min, a simple “topping-off” of glycogen stores to replenish muscle and liver glycogen lost during the prior day has been recommended typically with a CHO-rich diet of at least 6 g/kg [12] and up to 7–12 g/kg [8] in the 24 h period before the event. For events lasting >90 min however, glycogen supercompensation, or “carbo loading,” in the preceding 36–48 h may help improve performance by 2–3% [11]. Traditionally it had been recommended that in order to double glycogen stores in the classical supercompensation model [15], one had to exhaust glycogen stores with high-intensity exercise prior to high CHO intake. However recent studies show that short-term high-intensity exercise (or even complete physical inactivity) followed by a 1-day high (10–12 g/kg/day) intake of CHO similarly achieves glycogen supercompensation, and this is maintained for 3 days [11,16]. This latter point is particularly important to consider clinically, as it gives the athlete additional flexibility in athletes with gastrointestinal (GI) intolerability or GI distress prior to competition. In the final 1–4 h prior to the event, a single dose of 1–4 g/kg CHO is recommended for a final top-off of liver glycogen stores, as typically endurance events occur in the early morning directly after the overnight fast which depletes liver glycogen [8].

#### 3.1.2. During Competition, “Fueling”

For events lasting <60 min, no exogenous CHO ingestion is required [8,14]. However, for activities >60 min, active fueling strategies are recommended to maintain CHO accessibility. For events lasting 1–2.5 h, 30–60 g/h is commonly recommended [8,14] in a 6–8% CHO solution (concentrations typically found in commercial sports drinks) ideally consumed every 10–15 min [9] to maximally spare glycogen stores. For events lasting >2.5 h, higher CHO intakes of 60–70 g/h, and up to 90 g/h if tolerable are associated with improved performance [8]. This higher intake recommendation stems from research demonstrating that exogenous CHO oxidation peaks at a CHO ingestion rate of 1.0–1.1 g/min, due to the maximal GI absorption at this rate [11,17]. Including multiple CHO sources (glucose/fructose mixtures) at higher ingestion rates of 1.8 g/min can further increase oxidation up to 1.2–1.3 g/min due to differential intestinal transport mechanisms, and these glucose/fructose combinations also improve GI tolerance [8,11,12,17,18]. At these higher ends of intake, the authors recommend athletes routinely practice their fueling plan to assess GI comfort (e.g., liquid CHO may be more tolerable than solid) and practicality of their fueling plan. Fueling chances may vary according to rules of sport, e.g., halftimes during games, minimal/no fueling opportunity during swim portion of triathlon vs. ideal opportunity during bike, etc., and should be rehearsed. We also recommend athletes should practice their fueling plan at race/game intensity, as GI tolerability can be decreased on race day due to the increased stress response and sympathetic/parasympathetic imbalance on “game day.” Another important clinical consideration is in hot conditions; clinicians should counsel athletes to reduce CHO intake by 10% due to lowered CHO oxidation rates in hot environments [11].

In recent years, some athletes have manipulated their carbohydrate levels using a “train low” strategy involving lower intakes of CHO and higher intakes of fat. Periodically training in low glycogen/low glucose availability states may stimulate upregulation of fat oxidation pathways, spare glycogen stores, and may prolong time to exhaustion [12,19,20]. This low glucose state may be of advantage in ultra-endurance events where exercise is typically under 70% VO_2max_ and fuel sources are predominantly fats. Some athletes then decide to carbo load just prior to the event, so that they can in essence “train low, race high”: maximize both fat oxidation pathways at lower intensities (<70% VO_2max_) and glucose oxidation pathways at higher intensities (>70% VO_2max_). However, prolonged time spent in “train low” may reduce ability to generate maximal power in high-intensity situations [12,19]. In the authors’ clinical experience, “train low” may improve oxidative enzymes, but an athlete’s tolerability to maintain their training load decreases, and their quality of workouts and quality of their overall training stress (and therefore adaptation) declines. The hope is that by training in a low CHO state, the potential benefits from increasing fat oxidative enzyme pathways outweigh the negative effects of the lowered training load and training adaptations when racing in the high glucose state. In other words, “train low” may help improve an athlete’s “low gears” (maximizing fat oxidation) for prolonged exercise at lower intensities, but at the expense of losing the athlete’s “high gear” (maximal glucose oxidation) often needed during race situations. In addition, “train low” may adversely affect other types of training such as altitude training and consequently adaptation [21,22]. Furthermore, many of the “train low” studies are in a laboratory setting and not in a “real world” race situation. An interesting study by Cox et al. showed that while “train low” induced changes in mitochondrial enzyme activity (e.g., citrate synthase), there was no performance difference in actual exercise situations with either trained cyclists or triathletes involving steady-state exercise and time trial cycling [23]. Therefore, many suggest it could be a “tool in the tool belt” as part of an athlete’s overall training and nutrition plan but should not be employed in high-intensity training or race situations due to performance concerns [12,19].

Another recent technique is utilizing a CHO mouth rinse during endurance exercise [18,21] as a way to stimulate taste receptor cells and the central nervous system (CNS) to improve performance, without actual ingestion of CHO. A proposed mechanism of action is that it modulates the central governor theory [13], a CNS-established safe level of exertion during exercise to preserve an emergency reserve margin. The question was originally posed by Jeukendrup [24], who demonstrated time trial cycling performance improvements with glucose compared to placebo even in short-term (1 h) exercise. In his discussion, he concluded that it was unlikely CHO ingestion exerts its beneficial effect through its contribution to energy expenditure as only about 10–20% of ingested CHO is actually oxidized in the first hour of exercise, so “the explanation for this increased performance remains to be established.” Some proposed that it was simply the CHO presentation in the oral cavity that stimulated the CNS. A later study by Carter supports this showing that even an intravenous infusion of glucose during a 1 h time trial, despite increases in plasma glucose for oxidation and evidence of increased glucose uptake into the tissues, had no effect on 1-h cycling time trial performance [25]. A follow up study by the same group showed even a CHO mouth rinse (without ingestion) has a positive effect on 1 h time trial performance [26] and is likely mediated by CHO receptors in the mouth associated with CNS motivation pathways. A systematic review demonstrated that rinsing every 5–10 min (of at least 5–10 s of oral contact) with a 6.4–10% carbohydrate solution may improve performance by ~2–3% [21] in high intensity (>70% VO_2max_) exercises bouts of up to 1 h. We therefore suggest that for athletes with GI distress during high-intensity exercise that precludes actual oral carbohydrate intake, this strategy may be of value if the event is 1 h or less. However, any exercise of ~2 h or more, formal carbohydrate ingestion is imperative for performance [18] and only mouth rinsing without carbohydrate ingestion is not recommended.

In summary, daily CHO requirements vary according to level of exercise, from 5–7 g/kg/day (1 h/day of moderate exercise), 6–10 g/kg/day (1–3 h/day of exercise), to 8–12 g/kg/day (4≥ h/day of exercise). Pre-competition (“Loading”) recommendations also vary according to duration of exercise, from 6 g/kg/day (<90 min of exercise) to 10–12 g/kg/day (>90 min of exercise) with a 1–4 g/kg final “top-off” 1–4 h prior to event. During competition (“Fueling”) requirements similarly range from 30–60 g/h for <2.5 h of exercise, 60–70 g/h if >2.5 h of exercise, and up to 90 g/h for >2.5 h of exercise (if tolerable). As daily, pre-exercise, during exercise, and post-exercise CHO requirements are tiered to exercise level and can become confusing to the athlete, Table 1 provides a concise reference on the above CHO requirements. Post-exercise refueling of CHO is also a complex topic and separately discussed below in “Recovery Nutrition” and also outlined in Table 1.

### 3.2. Protein

Traditionally, endurance athletes have placed less of a priority on protein in comparison to carbohydrate. However, adequate protein intake and timing of intake are critical to any athlete, whether endurance or resistance trained. An outdated model is simply following nitrogen balance, which was originally designed to prevent nutrient deficiency, not optimize performance. Athletes require higher protein intakes [27] than the current Recommended Daily Allowance (RDA) of 0.8 g/kg/day in order to achieve training adaptations and improve performance [27,28].

#### 3.2.1. Daily Protein Requirements

The AND, DC, and ACSM all recommend protein ingestion for athletes in the range of 1.2–2.0 g/kg/day [8], with the ISSN recommending 1.4–2.0 g/kg/day [9]. Strength and power athletes are typically recommended to consume in the higher range and endurance athletes the lower range, based on individual needs. Temporary ingestion of higher quantities during intense training may provide additional benefit [9,27]. Muscle protein synthesis (MPS) is upregulated for 24 h following exercise and is due to the increased sensitivity to oral protein intake during this time [8,29]. This increased absorption provides an ideal time to optimize protein intake in order to maintain muscle mass after endurance exercise, as prolonged endurance exercise may induce a catabolic state and resultant muscle breakdown [8,9,30]. Timing and dose are also shown to be important; 0.25–0.3 g/kg of a quality protein source (see below) in the immediate 0–2 h post exercise provides approximately 10 g of essential amino acids (EAA) (which maximally stimulate MPS and the MPS associated signaling proteins mTOR, p70s6k, Akt needed for protein synthesis) [8,9,28,30]. Of note, either 0–2 h post-exercise or immediate pre-exercise protein intake both yield similar benefits (in non-ultra-endurance activities) [9,30]. Clinicians can educate athletes regarding this useful fact and let the decision be a matter of athlete preference and GI tolerance.

Athletes may think “more is better” and increase protein beyond recommendations. Daily intake of protein above the recommended level (1.2–2.0 g/kg/day and/or individual meals/doses beyond ~0.3 g/kg) have not been shown to be additionally beneficial, and MPS can only be stimulated with doses at least 3–5 h apart [8]. Temporary increases beyond 2.0 g/kg/day may be beneficial during short periods of intensified training beyond the athlete’s typical program, but routine higher total daily protein intake beyond this does not further benefit endurance athletes. In one study, 1.5 g/kg/day compared to 3.0 g/kg/day while keeping carbohydrate intake the same, did not result in improved endurance performance [4]. Therefore, the AND, DC, and ACSM recommend spreading protein dosing at ~0.3 g/kg every 3–5 h throughout the day [8].

#### 3.2.2. Pre-, During, and Post-Exercise Protein Requirements

Compared to resistance exercise, few studies have been done on pre- and during exercise protein intake with endurance activities, but available evidence shows it may improve same day and next day endurance performance [30]. In addition, importantly to competitive athletes, no studies have shown it hinders performance [30]. Exhaustive endurance exercise and significant eccentric activities e.g., marathons, downhill running, and obstacle course races can result in catabolism of muscle, especially in the setting of inadequate protein or reduced energy availability and does raise muscle creatine kinase levels (a marker of muscle damage) [8,9,11]. If tolerable, the athlete may therefore consider a pre-exercise dose of 0.3 g/kg protein according to GI tolerance. During endurance exercise (if particularly intense or significant eccentric exercise), approximately 0.25 g/kg protein per hour when taken along with carbohydrate is recommended by the ISSN to minimize potential muscle damage [9]. This can reduce creatine kinase elevations, improve subjective feelings of muscle soreness, and may increase MPS and net protein balance [9,11]. Post-exercise protein added to carbohydrate can increase muscle glycogen synthesis by 40–100% if in the setting of suboptimal post-exercise carbohydrate intake (i.e., <1 g/kg/h), however will not further increase glycogen synthesis if the athlete already has high carbohydrate intake (>1.2 g/kg/h) [11].

Traditionally, proteins containing branched-chain amino acids (BCAAs leucine, isoleucine, and valine) have garnered much attention in both popular media and research due to their role in protein metabolism, nerve function, and glucose/insulin regulation. However, in recent years, protein with higher EAA and leucine content (700–3000 mg) have emerged to be the ideal source to stimulate MPS [9]. Branched-chain amino acid supplementation still may help endurance athletes via central governor theory modulation [13]. BCAAs compete with tryptophan for transport across the blood brain barrier, and increased tryptophan may increase serotonin and contribute to feelings of fatigue [13]. However, BCAA supplements alone if not taken with a complete protein (i.e., adequate EAA content) may not adequately stimulate MPS [9]. Therefore, the authors suggest educating athletes on EAA (which contain BCAA) protein sources rather than solely BCAA which still pervade lay texts and popular media.

Many athletes (endurance and resistance athletes alike) continue to debate with passion their “go to” protein source, and part of the argument may relate to internal feelings and/or culture regarding their chosen diet (e.g., vegan, vegetarian, paleo, Mediterranean, flexitarian, the pesco-pollo-ovo-lacto vegetarian spectrum, etc.). From a scientific standpoint, dairy-based proteins (whey, casein and whole milk), lean meats, egg, and soy all stimulate MPS effectively [8]. However dairy-based proteins may be superior to other sources due to the higher leucine content and improved digestion/absorption kinetics of the EAAs found in liquid-based dairy foods [8].

In summary, protein doses of 0.3 g/kg (or ~20–40 g of protein covering the range of typical athlete builds), provides ~10–12 of EAA and ~1–3 g of leucine. When taken every 3–5 h spread throughout the day (including a dose immediately before or 0–2 h post-exercise) to a total of ~1.2–2.0 g/kg/day, this strategy may promote positive nitrogen balance and optimally benefit endurance athletes.

### 3.3. Fat

In comparison to carbohydrate, proper fat intake gathers less consideration by endurance athletes but is a worthy fuel source (oxidation of glycogen provides only ~2500 kilocalories of energy before depletion, whereas oxidation of fat provides at least 70,000–75,000 kilocalories of energy, even in a lean adult [31]). While the prototypical endurance athlete may prefer a carbohydrate-based diet due to the above explained benefits in the previous section, some ultra-endurance athletes have recently become interested in ketoadaptation (becoming “fat-adapted,” or “training low”) with a high fat, low carbohydrate diet [32]. This renewed interest is based on the higher oxidation of fat vs. glucose in lower intensity (<70% VO_2max_) exercise states typically seen in ultra-endurance events [19]. In the “train low” state of low carbohydrate availability, upregulation of lipid oxidation pathways does occur (such as citrate synthase and 3-hydroxyacyl-CoA dehydrogenase (3HAD)), albeit at the expense of carbohydrate metabolism downregulation [8,19,32]. If performance is not an issue, becoming fat-adapted and exercising at low (<70% VO_2max_) intensities therefore may improve lipolysis and promote weight loss in the overweight athlete. However, if the athlete’s focus is on racing and improving performance times, a high fat, low carbohydrate diet restricts that athlete’s ability to train and race and higher intensities [8] and may negatively affect their race outcome [8,12,19].

This is not to say that fat intake is irrelevant to athletes; fats are fundamental components of cell membranes, playing roles in signaling and transport, nerve function, providing insulation and vital organ protection, and are the source of essential dietary fatty acids [33]. Athletes who chronically restrict fat to <20% of total energy are at risk of low intake of fat-soluble vitamins and carotenoids, essential fatty acids including n-3 (omega-3) fatty acids [8,33], and possibly conjugated linoleic acids (CLA).

Conjugated linoleic acids are isomers of the essential n-6 linoleic acid, synthesized in the gut by bacteria and supplied in dairy products and ruminant meats (cow, sheep, goat, deer) [32]. Limited evidence shows that CLA may inhibit atherogenesis and carcinogenesis [32], important for athlete general health. Furthermore, and relevant to endurance athletes seeking to maintain body weight, CLA may reduce adipocyte uptake of lipids [32]. Knowledge of CLA’s effects on endurance exercise is currently limited and often conflicting, and most research has been on overweight subjects. In one placebo-controlled study, CLA at 0.9 g/day for 14 days significantly increased exercise time to exhaustion and tended to decrease perceived exertion in athletes [34], while 0.8 g/day for 8 weeks in another study showed no effect on time to exhaustion, VO_2max_, or body composition in healthy young men [35]. The ISSN acknowledges that while CLA animal studies are impressive, human studies are not yet convincing and currently considers CLA to have little evidence regarding supplementation [36].

Conjugated linoleic acids at higher doses (up to 6 g/day) and omega-3 rich fish oil supplementation may play a role in testosterone biosynthesis [27]. Proposed fish oil and CLA mechanism of action is to modulate CYP17A1 and HSD3B2 enzymes which decreases glucocorticoid metabolism and increases androgen pathway sex hormone metabolism [37]. This effect overall promotes an anabolic environment, important to endurance athletes who are susceptible to declines in testosterone seen with overtraining [37]. This point should especially be considered as a possible supplementation option in endurance athletes during periodization training at exceptionally high intensity training times or any athlete who is overreaching or overtrained and thereby risking testosterone suppression.

Medium-chain triglycerides (MCTs) have also gained attention in recent years, as MCTs can directly enter mitochondria and be used for energy via beta-oxidation [36]. This in theory provides the athlete with a readily available fat source for energy and thereby sparing glycogen [36]. While some studies suggest improved cycling performance with MCTs, other studies actually show ergolytic effects when taking MCTs versus carbohydrate, and furthermore most studies report GI complaints [36]; the ISSN currently considers MCTs in the category of “little to no evidence to support efficacy and/or safety” [36].

Endurance athletes are encouraged to follow public health guidelines to ensure adequate fat intake, and only consider limiting fat intake pre-race during a CHO loading phase or pre-race if there are GI comfort concerns [8]. Conjugated linoleic acids, fish oil, and MCTs may have promise, but further studies are needed to specifically define their role in endurance athletes.

### 3.4. Hydration

Fluid intake recommendations for endurance athletes have evolved [38,39,40]. Traditionally, athletes were told by coaches and other training staff that thirst is not a good indicator of hydration status. The theory espoused was that “once you’re thirsty you’re already dehydrated.” In a 1969 landmark study by Wyndham [41] assessing marathon participants in two endurance races, individuals who lost >2% of their body weight had elevated rectal temperatures, putting them at risk for hyperthermia. This prompted several individuals to suggest increasing fluid intake under the presumption that the thirst mechanism is an inadequate indicator of hydration. Interestingly however, the winner in both events of the Wyndham study actually had the highest overall rectal temperature and was asymptomatic at the end of the race. In retrospect this should have prompted further thought as not being as serious a health risk as believed. As late as 1996 the ACSM noted in their fluid replacement position stand [38] that “perception of thirst...cannot be used to provide complete restoration of water by sweating” and that “athletes should start drinking early and at regular intervals...or consume the maximal amount that can be tolerated.”

Consequently, athletes have historically tried to stay ahead of dehydration and drank before they were thirsty. However as clinical observations of overhydration mounted, the dangers became clearer. In the Boston marathon [42], an alarming 13% of finishers had hyponatremia which was even considered an underestimation, and 0.6% (90 finishers) were critical (≤120 mmol/L). Excessive consumption of fluids was the single most important risk factor in development of hyponatremia [42]. Lighter and slower runners are also at risk of positive fluid balance [43]. Exercise-associated hyponatremia (EAH) is used to describe hyponatremia occurring during or within 24 h after physical activity. It is defined by a serum, plasma, or blood sodium concentration below the reference laboratory range, which for most laboratories is <135 mmol/L [44]. Exercise-associated hyponatremia is serious and one of the potential causes of exercise-associated collapse [45] which can be fatal [42]. While sometimes asymptomatic, EAH can result in a myriad of signs and symptoms mimicking other conditions, including confusion, dyspnea, nausea, delirium, even coma and death [39,45]. In the authors’ clinical experience, it is often nonspecific and GI complaints may be the primary presenting symptom.

Noakes et al., helped bring EAH to the forefront with studies on ultra-endurance athletes demonstrating the pathophysiology of EAH including voluntary hyperhydration, increased sweat sodium loss, and loss of normal anti-diuretic hormone (ADH) suppression, called the syndrome of inappropriate ADH secretion (SIADH) [39,46]. Since the excretory capacity of the kidneys is ~800–1000 mL/h and fluid loss from exercise is estimated at an additional ~500 mL/h, in theory an athlete could conceivably consume up to 1.5 L/h without theoretical water retention [39]. However, dilution of serum sodium causing EAH commonly occurs at much lower water intake rates, placing the athlete at risk [39,45]. For practical recommendations, clinicians can explain to the athlete if he/she were to consume 1 L of fluid at rest, it would most likely simply be excreted with normally functioning kidneys. During exercise however, even small increases in ADH can markedly reduce kidney excretory capacity, thereby causing the athlete to retain fluid even if drinking less than 800–1000 mL/h [44]. The stimuli for SIADH include nausea/vomiting, hypoglycemia, hypotension, interleukin-6 (IL-6) release, and hyperthermia, all of which can occur with prolonged exercise [44,45]. Athletes are advised to monitor for SIADH stimuli e.g., nausea although as mentioned above, clinical symptoms of EAH may be nonspecific.

It was not until Noakes’ pivotal study in 2003 where the dangers of over-drinking were clearly described, and recommendations were updated [47]. The Advisory Statement by Noakes and the International Marathon Medical Directors Association suggest the athlete start with a hydration plan in the range of 400–800 mL/h [47], which was also adopted in the ACSM Position Stand in 2007 [40] recommending athletes drink ad libidum, in the suggested range of 400–800 mL/h. However, a hydration plan is individual to each athlete, and varies with sweat rates, sweat sodium content, intensity of exercise, body temperature and ambient temperature, bodyweight, kidney function, and many other factors. The ACSM suggests higher hydration rates for faster, heavier athletes competing in warm environments, and lower rates for slower, lighter athletes competing in cooler environments [40]. More specifically, a simulation study shows a 600 mL/h rate may be appropriate for a 70 kg athlete in cool or temperate (18 °C) running at speeds of 8.5–15 km/h [48]. However, it may produce overhydration in a 50 kg athlete running ≤10 km/h, or dehydration in a 90 kg athlete running ≥12.5 km/h. All athletes risk dehydration in warmer (28 °C) environments, however 50 kg athletes still risk overhydration at higher (800 mL/h) intakes and lower (≤12.5 km/h) speeds [48], further supporting that lighter slower runners are at increased risk and a hydration plan should be individualized [43].

Similarly, a sodium intake plan needs to be customized to an athlete’s experience, sweat rate and sweat sodium content, exercise intensity and environmental conditions. The AND, DC, and ACSM all recommend sodium ingestion during exercise in athletes with high sweat rates (>1.2 L/h), subjective “salty sweaters,” and prolonged exercise >2 h [9]. Although widely variable, average sweat rates range from 0.3 to 2.4 L/h [9] and the average sodium sweat content is 1 g/L (50 mmol/L) [9]. A sports drink containing sodium in the range of 10–30 mmol/L (230–690 mg/L) results in optimal absorption and prevention of hyponatremia [11], a concentration found in typical commercial sports drinks. The ACSM recommendations for sodium intake during exercise is to start with ~300–600 mg/h (1.7–2.9 g salt) during a prolonged exercise bout and adjust intake accordingly [36]. Post-exercise fluid and sodium repletion recommendations are discussed in Recovery Nutrition section below.

Therefore, following the instinctive thirst mechanism and monitoring bodily parameters such as body weight, urine color, race pace, body temperature, and environmental temperature with each workout can help the athlete fine tune their individual hydration needs and avoid complications of EAH [19]. Further advice for the overdrinking athlete could include presenting the dichotomy illustrated in the 2007 ACSM position stand: while dehydration can impair exercise performance and contribute to heat illness or exacerbate exertional rhabdomyolysis, exercise-associated hyponatremia can produce grave illness or even death [40].

### 3.5. Supplements and “Hot Topics”

#### 3.5.1. Nitrates

Dietary nitrate has been used for years in medical conditions such as cardiovascular disease and hypertension [49]. It has gained significant attention in the endurance athlete population after a pivotal 2007 study [50] by Larsen that showed a decreased oxygen cost for submaximal exercise workloads. Since then several publications have surfaced: a PubMed search on “nitrate supplementation exercise” yielded only 52 publications in the previous 10 years (2004–2013), but over 180 publications in the last 5 years (2014–2018). Certain vegetables such as beets, and beetroot juice, contain high levels of inorganic nitrate (NO3^−^). Once consumed, NO3^−^ is converted to NO2^−^ by oral bacteria, and then to nitric oxide (NO) in the gut [51,52]. Nitric oxide has numerous bodily effects relevant to endurance athletes, ranging from vasodilation, blood flow and O_2_ regulation in working muscle, mitochondrial respiration and biogenesis, glucose uptake, and overall muscle contraction/relaxation [49,51,52]. Cumulatively, these effects can improve muscle economy, efficiency and mitigate fatigue, positively impact cardiorespiratory performance by decreasing effort at submaximal workloads, and in some studies improve time trial performance (albeit mainly in non-elite athletes) [12,51,52,53].

Beetroot juice specifically (compared to other forms of dietary nitrate) has been studied in athletes. If taken up to 2–3 h prior to endurance exercise it can reduce oxygen cost during exercise, may improve time to exhaustion, cardiorespiratory performance at anaerobic threshold, and VO_2max_ [45]. Results however are currently mixed and sometimes contradictory; many positive studies are on subjects of 10 or fewer, and effects may be less pronounced or not even benefit already trained/elite athletes due to their nutrition plan (already containing adequate nitrate) and/or improved metabolic efficiency from maximizing training adaptations [12,53]. Furthermore, multi-day high nitrate intake or supplementation may help raise nitrate levels and improve performance compared to a control diet. In one study, 6 days of a high nitrate diet (8.2 mmol/day from vegetables and fruits) compared to a control diet (2.9 mmol/day) induced a significant rise in plasma nitrate and was associated with a reduced oxygen cost during moderate intensity cycling, higher muscle work during high-intensity fatiguing leg exercise, and improved performance during repeated sprints [54]. This study may help further explain the variability in results with acute single-day supplementation, and help the athlete target healthy nitrate intake levels.

Dosing also varies, and in studies typically ranges from either 300–600 mg of nitrate supplement and up to 10 mg/kg, 0.1 mmol/kg with minimum 6–8 mmol total, 500 mL of beetroot juice, or approximately 3–6 whole beets [12,36,51]. Timing may also play a role in the variability of results. Recent data show that beetroot juice consumption should ideally commence within 90 min of exercise rather than 2–3 h prior as in earlier studies, since NO levels peak at 2–3 h and then sharply fall leaving the athlete in a potentially suboptimal time interval for exercise [51,55,56]. Manner of ingestion must also be considered when interpreting study results. Mouth rinse, oral antiseptics, or limited nitrate supplement oral contact time can all limit NO3^−^ to NO2^−^ conversion [12,19,51]. Since 500 mL of beetroot juice pre-race can have significant GI distress in some athletes (and may contribute to overhydration), beetroot juice concentrate, powders, and “shots” have been commercially developed and may be an option.

A few commonly missed practical points are worth mentioning. One is the significant expense of commercial nitrate or beet supplements. Athletes could consider just eating sufficient high-nitrate vegetables or actual beets which supply similar levels of nitrates. In the Larsen study the daily nitrate dose used were in amounts achievable through a diet rich in vegetables, specifically “the amount normally found in 150 to 250 g of a nitrate-rich vegetable such as spinach, beetroot, or lettuce” [50]. Additionally, in the authors’ experience nitrate supplements may become rancid if left out and powders can easily harden with moisture rendering scooping impossible, so supplements should be tightly sealed, refrigerated and kept out of direct light. Athletes should be alerted to the possibility of beeturia and red bowel movements, which is normal [52]. Lastly, dietary nitrate supplements also mildly lower diastolic and mean arterial blood pressure [57], which may be an issue in those with low blood pressure, orthostasis, or at risk for hypotension.

#### 3.5.2. Antioxidants

The role of antioxidant supplements in sport was notably questioned by Gomez-Cabrera [58,59,60,61] who highlighted the potential blunting of the training adaptation response to exercise. Consuming high doses of single antioxidants (such as vitamins C and E) may inhibit the signaling pathways normally triggered by the oxidative stress of exercise during training. The pro-oxidant environment including the buildup of reactive oxygen species (ROS) from exercise triggers adaptations in the form of increased superoxide dismutase and glutathione peroxidase enzymes, muscle repair, and mitochondrial biogenesis pathways [58,59,60,61,62]. While a healthy diet for athletes should naturally include a variety of antioxidants, supraphysiologic high doses of single antioxidants may impair or prevent training adaptations in endurance athletes and are not recommended. However, once an endurance athlete has already peaked in training, and their main goal is timely recovery, a food or supplement containing a variety of antioxidants (e.g., dark berries, dark leafy greens) may help to speed recovery and return to competition [62]. In a review, tart cherry juice at 8–12 oz twice a day (or 1 oz if concentrate), taken 4–5 days prior and 2–3 days after an event may promote recovery [62]. This may be useful for multi-day endurance events such as cycling tours, multi-stage races, etc.

Green tea contains various bioactive phytochemicals, including high levels of the antioxidant polyphenols epigalocatechin gallate (EGCG), catechin, epicatechin, epigalocatechin, and epicatechin gallate (commonly known as catechins). The proposed health benefits of green tea are typically attributed to its antioxidant properties that can scavenge ROS and free radicals associated with many chronic diseases [63,64]. Relevant to endurance athletes, green tea extracts have been shown to stimulate fat oxidation and weight loss at ranges of 270–1200 mg/day [63]. The catechins work as a catechol-o-methyltransferase (COMT) inhibitor (potentiating effects of norepinephrine, thermogenesis and fat oxidation) and phosphodiesterase inhibitor (preventing breakdown of cyclic adenosine monophosphate (cAMP) which stimulates hormone-sensitive lipase) [63]. In a review, green tea extract was shown to enhance fat oxidation and improve performance during endurance exercise [65]. Furthermore, green tea extract’s effect is more pronounced with additional caffeine supplementation [65,66]. Endurance athletes looking to maximize fat oxidation and spare glycogen during longer, lower intensity events may find this valuable. Asian populations have higher concentrations of high-activity COMT polymorphism than Caucasians, and so there may be a population-specific effect in Asian populations [67]. Of note, few human studies exist regarding green tea extract on athletic performance. Commonly referenced studies that claim an 8–24% increase in time to exhaustion with swimming [68] and a 30% increase in time to exhaustion with running [69] due to increased fat oxidation, while very impressive results, were actually animal studies. In a review documenting later studies in humans, green tea has not shown a similar performance enhancement [63], and the few studies that did suggest improvements were only in untrained sedentary populations. Therefore, it remains to be seen if green tea catechins exert a significant performance effect on trained and non-overweight athletes. Additionally, a final word of warning bears consideration: since the methods of growing, harvesting, and preparing green tea vary widely (and any supplement may contain contaminants or banned substances), athletes should exercise caution. When selecting green tea, green tea extract, or any other supplement, the authors advocate to choose wisely from trusted sources (e.g., Institute of National Anti-Doping Organizations, Council for Responsible Nutrition, UL^®^, Informed-Choice.org) [70].

#### 3.5.3. Caffeine

Caffeine, a popular supplement in the general population, has been heavily researched in sports for its ergogenic effects. Caffeine is a trimethylxanthine, similar to adenosine in chemical structure [71]. It has numerous proposed mechanisms of action. Centrally, by blocking adenosine receptors in the CNS it acts as a stimulant to increase neurotransmitter release [72,73], increases cognitive performance [72], and suppresses pain by increasing β-endorphins [72,73]. Peripherally, caffeine increases motor unit recruitment [73] and helps mobilize calcium to increase muscle contraction [72]. Systemically, caffeine assists in mobilization of fatty acids for energy (decreasing dependence on glycogen) and increases thermogenesis [72]. There is good consensus on dosage and timing; meta-analyses and reviews [72,73] all recommend a moderate caffeine dose of 3–6 mg/kg 30–90 min prior to exercise to maximize effects. This dosing can improve sustained maximal endurance (e.g., time trial) performance and vigilance during endurance tasks [72]. Of additional practical importance is the synergistic effect of caffeine when consumed with carbohydrate. Taking the two together improves cycling work production compared to caffeine or carbohydrate alone, while perception of work remains unchanged [74]. Of note in one study, the anhydrous form of supplemental caffeine may have a greater ergogenic effect than drinking coffee [75], although in this study the caffeine capsule was actually taken with water. Therefore, the authors question if practically this ultimately may be similar to drinking coffee.

Higher caffeine doses of 9 mg/kg do not further enhance performance [72,76], and can result in very undesirable side effects including GI distress, nervousness, confusion, disturbed sleep [77]. Athletes may also be concerned about severe or systemic side effects with this level of dosing. A recent review proposes that caffeine does not result in more serious complications such as water-electrolyte imbalances, dehydration, hyperthermia, or reduced exercise-heat tolerance [78]. However, doses above 9 mg/kg may result in urinary caffeine detection and considered above the doping threshold in many professional sports organizations [36]. Fortunately, lower doses <3 mg/kg can be similarly ergogenic in endurance cycling and running research, improving vigilance, mood, and cognitive function, without any major side effects [77].

Traditionally, there has been a long-held paradigm that habitual caffeine intake may blunt the ergogenic effects of acute pre-exercise caffeine consumption. Research has shown that the performance benefit of caffeine lessens after 15–18 days of daily low dose (3 mg/kg) caffeine ingestion during peak cycling power in Wingate and incremental exercise cycling tests [79]. By 4 weeks at the same 3 mg/kg dose the cycling performance benefit was no longer apparent in time trial performance [80]. However other research has shown that athletes with low, moderate, or even high habitual caffeine intake exhibit similar absolute and relative improvements in cycling time-trial performance to an acute 6 mg/kg caffeine dose [81].

Furthermore, caffeine may also be helpful during prolonged (>2 h) exercise. In one study, upon a 2 h cycling at 60% VO_2max_ interspersed with bouts of high-intensity (82% VO_2max_) exercise followed by a cycling time-trial, athletes were given either low dose (1.5 mg/kg) or moderate dose (2.9 mg/kg) caffeine at 80 min in to the cycling challenge. Both caffeine groups had faster time-trial completion compared to placebo, and the moderate dose group had improved performance to a greater extent than the low dose group [82]. This study suggests the concept of “topping-up” of caffeine periodically during prolonged exercise, and therefore may also be helpful for ultra-endurance events.

The authors recommend that athletes begin at lower doses if they are not caffeine tolerant and adjust accordingly. Some athletes find it useful to cycle caffeine with periods of abstaining from coffee during lower intensity training or prior to races, then resuming coffee at race time or during high intensity training. A safe starting dose may be up to 3 mg/kg. With daily caffeine intake, the performance benefit begins to decline at about 15–18 days and may disappear by 4 weeks. For habitual coffee drinkers an acute 6 mg/kg supplementation may be an option on race day if tolerated. Periodic topping-up during prolonged exercise (authors suggest every 1–2 h as needed) may also be of benefit.

#### 3.5.4. Probiotics

Probiotics are considered “live food ingredients” that provide a beneficial effect to the host organism [83] and occur naturally in fermented foods such as yogurt, kimchee, sauerkraut, miso and natto, or can be taken in supplement form. Most commonly *Lactobacillus* and *Bifidobacteria* are the primary species used [83] and produce lactic acid from carbohydrates to provide the sour taste in fermented foods. Probiotics have many proposed health benefits including antimicrobial activity to improve diarrheal illness and reduce urogenital infection, assisting with lactose intolerance, preventing constipation, improving immune function, and possibly even having anticarcinogenic effects to the colon [83]. Endurance athletes are susceptible to upper respiratory infection (URI), and elite athletes have a higher rate of URI than recreational athletes [84]. Probiotics may play a role in reducing these symptoms [85]. The field of probiotic research in athletes is still in its early stages, and few studies exist regarding performance outcomes [86]. In a review of the literature, only six studies were found, and while two did show an ergogenic effect on performance, one study was in mice [86].

A recent review in healthy physically active people and athletes showed probiotics may help with reduction of GI and upper respiratory symptoms [85]. Endurance athletes when fatigued show similar clinical characteristics of patients who experience reactivation of Epstein Barr virus (less T-cell secretion of interferon gamma) and exhibit diminished natural killer cell activity [83]. Probiotic supplementation can improve mucosal T-cell interferon concentration to normal levels and attenuate the reduction in natural killer cell activity [83]. The probiotic strain, dose, period of consumption and form of administration however (e.g., capsules, probiotic sachets, fermented food) likely play a role in its effect; multiple strain probiotics, in the form of fermented food or sachet when taken for a longer compared to shorter periods of time appear to show better results [85]. These benefits may help the athlete in terms of comfort and recovery from exercise, and therefore indirectly may play a role in performance. Endurance athletes prone to URI or GI symptoms, those susceptible to infection, or who travel frequently for events and are exposed to travel-related illness, may especially find benefit.

#### 3.5.5. Recovery Nutrition

A note should be made on the topic of recovery, as many athletes may not know of the post-exercise nutritional “window of opportunity” to facilitate a timely recovery to pre-exercise status. Carbohydrate and water have the most research, but the role of post-exercise protein, caffeine, and antioxidants may have important impacts on endurance athletes. Studies have shown that high carbohydrate (8–10 g/kg/day) refeeding can restore pre-exercise glycogen values within 24 h [11]. Aggressive carbohydrate refeeding at 1.2 g/kg/h for the first few hours post-exercise should be implemented if glycogen repletion is needed quickly for another event with <4 h recovery time [11,30]. In these situations, high glycemic index foods (>70) are preferred [30]. Ideally, dosing at 15–30-min intervals achieve the highest glycogen synthesis rates in the early first 3–5 h recovery period [11]. If an athlete cannot tolerate this carbohydrate volume, the addition of caffeine (3 mg/kg, even up to 8 mg/kg if no side effects) can boost glycogen repletion up to 66% more [30,87]. If the athlete can only tolerate 0.8 g/kg/h of carbohydrate, adding protein at 0.2–0.4 g/kg/h can also boost glycogen repletion [30,87]. Adding protein to carbohydrate intakes of ≥1.2 g/kg/h does not further improve glycogen synthesis however [11]. If the exercise had a significant eccentric component leading to significant muscle damage (marathons, downhill running), post-exercise protein with a high leucine content (700–1300 mg) within the first 2 h can stimulate MPS and recovery [9,30]. Similarly, eccentric and highly stressful exercise bouts that raise free radical and ROS levels can delay recovery to peak form due to the excessive oxidant load surpassing the innate antioxidant system [58,59,60,61]; ingestion of high antioxidant foods such as tart cherry juice may improve recovery [62].

Long endurance bouts can challenge hydration status; fluid and therefore bodyweight loss is expected with exercise and 0.1–3.0% bodyweight loss after exercise is still defined as euhydration [44,45]. Typical recommendations are to replace fluid with 150% of fluid lost based on bodyweight, and more of this fluid is retained when there is moderate to high sodium content (>60 mmol/L) [11]. This translates roughly to >1380 mg of sodium/L, amounts significantly higher than in typical sports drinks. Some athletes turn to salt packets or tablets, especially if they are “salty sweaters,” training in hot environments, or have a history of exercise-associated muscle cramps [12]. However, the authors caution this strategy in hypertensives or those needing to restrict total sodium intake. Interestingly, addition of potassium does not show any additional rehydration benefit [11].

#### 3.5.6. Concern with High Carbohydrate Diets?

There has been considerable discussion in both the medical literature and popular media [88,89] regarding the recent trend of low carbohydrate diets. Much of this concern may stem from the obesity epidemic, the high carbohydrate content and lack of adequate fruit and vegetable intake of typical Western diets, and the lack of adequate physical activity/sedentary lifestyle of well-developed countries [90]. This leads to an overabundance of fuel in the form of carbohydrate and difficulties with “glucose disposal” in the absence of adequate exercise to “burn the excess fuel.” Additionally, continual exposure to elevated blood glucose levels may lead to neurodegeneration [89]. Dementia has even been colloquially referred to as “type 3 diabetes.” Athletes may worry what potential effects consuming high glycemic meals or foods have on training-related metabolic responses and exercise performance. This very question was addressed in the joint position statement by the AND, DC, and ACSM: there is “Grade I—Good evidence” that neither the glycemic load nor glycemic index of carbohydrate-rich meals affects metabolic or performance outcomes when conditions are matched for carbohydrate and energy content [8].

Nevertheless, endurance athletes may find it useful to purposefully exercise in lower carbohydrate availability states. This may not only be for health concerns but as part of a periodization training plan, to enhance the training stimulus, or even improve mental toughness and “grit” [91] by exercising in low carbohydrate states. “Grit” is a concept of mental toughness developed by Angela Duckworth [91] that describes one’s passion and perseverance toward a goal and holding steadfast to that goal despite barriers. Endurance athletes may experience this when exercising in difficult conditions, such as bad weather and low fuel availability. Exercising in low carbohydrate states can be achieved in several ways: for example, reducing total carbohydrate intake at certain times of their training plan, training in a fasted state, performing two training sessions in close proximity without adequate refueling, or simply early morning training before breakfast [8]. Since mental toughness can play a key role in performance [91], the nutritional variables above could be considered by endurance athletes and incorporated into an athlete’s mental training.

## 4. Discussion

The world of endurance nutrition is continually evolving, and clinicians need to keep up-to-date as research emerges on sports nutrition topics. Furthermore, the commercial supplement industry is ever-changing. New products with purported benefits are being advertised to athletes as well as the general population, claiming improvements in performance and general health. Elite athletes, after they maximize training adaptations, often look to gain marginal benefits that they believe may be pivotal in whether they make the podium or not. They may take supplements and/or adopt new diets faster than medical research can keep up to make sound recommendations. This paper therefore summarizes the latest evidence-based recommendations as well as highlights hot topics and controversial subjects in order to equip the clinician dealing with the endurance athlete with current knowledge. Table 1 summarizes the key recommendations for macronutrients, hydration, and supplements.

High carbohydrate diets have long been tested and continues to be recommended in endurance athletes. Most endurance athletes are familiar with high carbohydrate diets, but the importance of protein (both total daily intake and immediate post-exercise consumption) may not be as well-known by athletes. Attention to adequate intake is emphasized to improve recovery, ameliorate muscle damage, and maintain muscle mass. Fats recently have been gaining popularity again, especially with ultra-endurance athletes, although “training low” with a high-fat diet may not necessarily improve performance. There has been a shift away from forced hydration plans and personalizing fluid intake according to thirst and sweat rates to avoid exercise-associated hyponatremia.

Athletes commonly take supplements, and a few supplements may have merit in the endurance world. Nitrates may help reduce oxygen cost and improve time to exhaustion, possibly cardiorespiratory performance at anaerobic threshold, and even VO_2max_. Studies are mixed however, and nitrate may preferentially benefit non-elite recreational athletes. Antioxidants may help an athlete who has already peaked in terms of training adaptation, where the main goal is facilitating recovery and earlier return to competition in multi-stage events. Caffeine has a very large body of research behind its ergogenic effects, with side effects being the main limiting factor. There is a paucity of quality research on probiotics for athletes, but chronic URI and GI symptoms common in endurance athletes may potentially be attenuated with *Lactobacillus* and *Bifidobacteria* supplementation. Additionally, as with any supplement, since the US Food and Drug Administration (FDA) is not authorized to review dietary supplement products for safety and effectiveness before they are marketed, there is the risk of contaminants and illicit substances in commercial supplements. These substances may not only present a safety risk but may be on a banned substance list for professional athletes [92]. While it is recommended that athletes obtain nutrition from whole foods, we acknowledge that athletes may take supplements and recommend they choose from trusted sources.

We hope this review helps clinicians treating and counseling endurance athletes clear up misconceptions athletes may have regarding sports nutrition and provide evidence-based recommendations according to current research. In the absence of high-quality evidence, we also provide practical recommendations on select supplements and offer our clinical advice on specific topics based on years of treating endurance athletes. Future research may help shed new light on the potential pleotropic benefits of probiotics, fats including CLA, fish oil, and MCTs, more clarity of the roles of nitrates and antioxidants, and the ideal balance of low vs. high carbohydrate intake to optimize both general athlete health and athletic performance.

## Figures and Tables

**Table 1:** Key recommendations for macronutrients, hydration, and supplements (exercise duration is listed in italics within parentheses).

Nutrient	Daily Requirements	Pre-Exercise	During Exercise	Post-Exercise
Carbohydrate	5–7 g/kg/day *(1 h/day)* 6–10 g/kg/day *(1–3 h/day)* 8–12 g/kg/day *(4≥ h/day)*	6 g/kg/day *(<90 min)* 10–12 g/kg/day *(> 90 min) +* 1–4 g/kg (1–4 h prior to event)	30–60 g/h *(<2.5 h)* 60–70 g/h *(>2.5 h)* 90 g/h *(>2.5 h, if tolerable)*	8–10 g/kg/day (first 24 h) 1.0–1.2 g/kg/h (first 3–5 h) or 0.8 g/kg/h + protein (0.3 mg/kg/h) or caffeine (3 mg/kg)
Protein	1.4 g/kg/day 0.3 g/kg every 3–5 h	0.3 g/kg immediately prior (or post–exercise)	0.25 g/kg/h (if high intensity/eccentric exercise)	0.3 g/kg within 0–2 h (or pre-exercise)
Fat	Do not restrict to <20% total caloric energy Unclear role of CLA, omega-3, MCT supplements Consider limiting fat intake only during carbohydrate loading, or pre-race if GI comfort concerns			
Water	Try initial hydration plan at ~400–800 mL/h; Adjust according to individual athlete variations (sweat rates, sweat sodium content, exercise intensity, body temperature, ambient temperature, bodyweight, kidney function) Follow thirst mechanism, monitor parameters (bodyweight, urine color)			Replace fluid with 150% of fluid lost
Sodium	Try initial sodium plan at 300–600 mg/h if high sweat rate (>1.2 L/h), subjective “salty sweater,” or prolonged exercise >2 h Adjust intake according to individual athlete variations (sweat rates, sweat sodium content, exercise intensity, body temperature, ambient temperature, bodyweight, kidney function)			Improved water repletion observed with >60 mmol/L sodium content (~1380 mg/L)
Nitrates	300–600 mg of nitrate (up to 10 mg/kg or 0.1 mmol/kg) or 500 mL beetroot juice or 3–6 whole beets within 90 min of exercise onset Consider multi-day dosing e.g., 6 days of a high-nitrate diet prior to event			
Antioxidants	Avoid prior to exercise to maximize training adaptation Take prior to exercise only if recovery needed within 24 h Many options: whole foods, dark berries, dark greens, green tea e.g., 8–12oz tart cherry juice twice a day (1oz if concentrate) 4–5 days prior and 2–3 days after event e.g., green tea extract (270–1200 mg/day)			
Caffeine	3–6 mg/kg taken 30–90 min prior to exercise Consider “topping-up” every 1–2 h as needed ≥9 mg/kg does not further enhance performance, may have undesirable side effects, + drug test ≤3 mg/kg can also be ergogenic without side effects			3 mg/kg with carbohydrate enhances glycogen repletion
Probiotics	*Lactobacillus* and *Bifidobacteria* may help with upper respiratory and/or GI symptoms			

## References

[1] (2017). Behind USAT and Ironman’s Efforts to Grow the Sport. https://www.triathlete.com/2017/03/lifestyle/behind-usat-ironmans-efforts-grow-sport_299133.

[2] Shilton A. Let’s Try a Triathlon—The New York Times. https://www.nytimes.com/guides/well/triathlon-training.

[3] Miller J.A. The Running Bubble Has Popped. (You Couldn’t Hear It in New York.)—The New York Times. https://www.nytimes.com/2017/11/05/sports/ny-marathon-running.html.

[4] Costa R.J.S., Hoffman M.D., Stellingwerff T. (2019). Considerations for Ultra-Endurance Activities: Part 1—Nutrition. Res. Sports Med..

[5] Nikolaidis P.T., Veniamakis E., Rosemann T., Knechtle B. (2018). Nutrition in Ultra-Endurance: State of the Art. Nutrients.

[6] Ebell M.H., Siwek J., Weiss B.D., Woolf S.H., Susman J., Ewigman B., Bowman M. (2004). Strength of Recommendation Taxonomy (SORT): A Patient-Centered Approach to Grading Evidence in the Medical Literature. AFP.

[7] U.S. Department of Health & Human Services, Agency for Healthcare Research and Quality Clinical Guidelines and Recommendations. https://www.ahrq.gov/professionals/clinicians-providers/guidelines-recommendations/index.html.

[8] Jäger R., Kerksick C.M., Campbell B.I., Cribb P.J., Wells S.D., Skwiat T.M., Purpura M., Ziegenfuss T.N., Ferrando A.A., Arent S.M. (2017). International Society of Sports Nutrition Position Stand: Protein and Exercise. J. Int. Soc. Sports Nutr..

[9] Thomas D.T., Erdman K.A., Burke L.M. (2016). Position of the Academy of Nutrition and Dietetics, Dietitians of Canada, and the American College of Sports Medicine: Nutrition and Athletic Performance. J. Acad. Nutr. Diet..

[10] Spriet L.L. (2014). New Insights into the Interaction of Carbohydrate and Fat Metabolism during Exercise. Sports Med..

[11] Jeukendrup A.E., Jentjens R.L.P.G., Moseley L. (2005). Nutritional Considerations in Triathlon. Sports Med..

[12] Getzin A.R., Milner C., Harkins M. (2017). Fueling the Triathlete: Evidence-Based Practical Advice for Athletes of All Levels. Curr. Sports Med. Rep..

[13] Noakes T.D. (2000). Physiological Models to Understand Exercise Fatigue and the Adaptations That Predict or Enhance Athletic Performance. Scand. J. Med. Sci. Sports.

[14] Burke L.M., Hawley J.A., Wong S.H.S., Jeukendrup A.E. (2011). Carbohydrates for Training and Competition. J. Sports Sci..

[15] Bergström J., Hermansen L., Hultman E., Saltin B. (1967). Diet, Muscle Glycogen and Physical Performance. Acta Physiol. Scand..

[16] Bussau V.A., Fairchild T.J., Rao A., Steele P., Fournier P.A. (2002). Carbohydrate Loading in Human Muscle: An Improved 1 Day Protocol. Eur. J. Appl. Physiol..

[17] Jeukendrup A.E., Moseley L., Mainwaring G.I., Samuels S., Perry S., Mann C.H. (2006). Exogenous Carbohydrate Oxidation during Ultraendurance Exercise. J. Appl. Physiol..

[18] Jeukendrup A. (2014). A Step Towards Personalized Sports Nutrition: Carbohydrate Intake During Exercise. Sports Med..

[19] Getzin A.R., Milner C., LaFace K.M. (2011). Nutrition Update for the Ultraendurance Athlete. Curr. Sports Med. Rep..

[20] Hansen A.K., Fischer C.P., Plomgaard P., Andersen J.L., Saltin B., Pedersen B.K. (2005). Skeletal Muscle Adaptation: Training Twice Every Second Day vs. Training Once Daily. J. Appl. Physiol..

[21] Silva T.d.A.e., de Souza M.E.D.C.A., de Amorim J.F., Stathis C.G., Leandro C.G., Lima-Silva A.E. (2013). Can Carbohydrate Mouth Rinse Improve Performance during Exercise? A Systematic Review. Nutrients.

[22] Jeukendrup A.E., Chambers E.S. (2010). Oral Carbohydrate Sensing and Exercise Performance. Curr. Opin. Clin. Nutr. Metab. Care.

[23] Cox G.R., Clark S.A., Cox A.J., Halson S.L., Hargreaves M., Hawley J.A., Jeacocke N., Snow R.J., Yeo W.K., Burke L.M. (2010). Daily Training with High Carbohydrate Availability Increases Exogenous Carbohydrate Oxidation during Endurance Cycling. J. Appl. Physiol..

[24] Jeukendrup A., Brouns F., Wagenmakers A.J., Saris W.H. (1997). Carbohydrate-Electrolyte Feedings Improve 1 h Time Trial Cycling Performance. Int. J. Sports Med..

[25] Carter J.M., Jeukendrup A.E., Mann C.H., Jones D.A. (2004). The Effect of Glucose Infusion on Glucose Kinetics during a 1-h Time Trial. Med. Sci. Sports Exerc..

[26] Carter J.M., Jeukendrup A.E., Jones D.A. (2004). The Effect of Carbohydrate Mouth Rinse on 1-h Cycle Time Trial Performance. Med. Sci. Sports Exerc..

[27] Phillips S.M., Van Loon L.J.C. (2011). Dietary Protein for Athletes: From Requirements to Optimum Adaptation. J. Sports Sci..

[28] Phillips S.M. (2012). Dietary Protein Requirements and Adaptive Advantages in Athletes. Br. J. Nutr..

[29] Burd N.A., West D.W.D., Moore D.R., Atherton P.J., Staples A.W., Prior T., Tang J.E., Rennie M.J., Baker S.K., Phillips S.M. (2011). Enhanced Amino Acid Sensitivity of Myofibrillar Protein Synthesis Persists for up to 24 h after Resistance Exercise in Young Men. J. Nutr..

[30] Kerksick C.M., Arent S., Schoenfeld B.J., Stout J.R., Campbell B., Wilborn C.D., Taylor L., Kalman D., Smith-Ryan A.E., Kreider R.B. (2017). International Society of Sports Nutrition Position Stand: Nutrient Timing. J. Int. Soc. Sports Nutr..

[31] Wilmore J.H., Costill D.L., Kenney W.L., Wilmore J.H., Costill D.L., Kenney W.L. (2008). Fuel for Exercising Muscle: Metabolism and Hormonal Control. Physiology of Sport and Exercise.

[32] Volek J.S., Noakes T., Phinney S.D. (2015). Rethinking Fat as a Fuel for Endurance Exercise. Eur. J. Sport Sci..

[33] Institute of Medicine, Food and Nutrition Board, Institute of Medicine (U.S.) (2005). Total fat and fatty acids. Dietary Reference Intakes for Energy, Carbohydrate, Fiber, Fat, Fatty Acids, Cholesterol, Protein, and Amino Acids.

[34] Terasawa N., Okamoto K., Nakada K., Masuda K. (2017). Effect of Conjugated Linoleic Acid Intake on Endurance Exercise Performance and Anti-Fatigue in Student Athletes. J. Oleo Sci..

[35] Tajmanesh M., Aryaeian N., Hosseini M., Mazaheri R., Kordi R. (2015). Conjugated Linoleic Acid Supplementation Has No Impact on Aerobic Capacity of Healthy Young Men. Lipids.

[36] Kerksick C.M., Wilborn C.D., Roberts M.D., Smith-Ryan A., Kleiner S.M., Jäger R., Collins R., Cooke M., Davis J.N., Galvan E. (2018). ISSN Exercise & Sports Nutrition Review Update: Research & Recommendations. J. Int. Soc. Sports Nutr..

[37] Macaluso F., Barone R., Catanese P., Carini F., Rizzuto L., Farina F., Di Felice V. (2013). Do Fat Supplements Increase Physical Performance?. Nutrients.

[38] Convertino V.A., Armstrong L.E., Coyle E.F., Mack G.W., Sawka M.N., Senay L.C., Sherman W.M. (1996). American College of Sports Medicine Position Stand. Exercise and Fluid Replacement. Med. Sci. Sports Exerc..

[39] Noakes T.D., Sharwood K., Speedy D., Hew T., Reid S., Dugas J., Almond C., Wharam P., Weschler L. (2005). Three Independent Biological Mechanisms Cause Exercise-Associated Hyponatremia: Evidence from 2,135 Weighed Competitive Athletic Performances. Proc. Natl. Acad. Sci. USA.

[40] American College of Sports Medicine Position Stand Exercise and Fluid Replacement. -PubMed-NCBI. https://www.ncbi.nlm.nih.gov/pubmed/17277604.

[41] Wyndham C.H., Strydom N.B. (1969). The Danger of an Inadequate Water Intake during Marathon Running. S. Afr. Med. J..

[42] Almond C.S.D., Shin A.Y., Fortescue E.B., Mannix R.C., Wypij D., Binstadt B.A., Duncan C.N., Olson D.P., Salerno A.E., Newburger J.W. (2005). Hyponatremia among Runners in the Boston Marathon. N. Engl. J. Med..

[43] Chorley J., Cianca J., Divine J. (2007). Risk Factors for Exercise-Associated Hyponatremia in Non-Elite Marathon Runners. Clin. J. Sport Med..

[44] Hew-Butler T., Ayus J.C., Kipps C., Maughan R.J., Mettler S., Meeuwisse W.H., Page A.J., Reid S.A., Rehrer N.J., Roberts W.O. (2008). Statement of the Second International Exercise-Associated Hyponatremia Consensus Development Conference, New Zealand, 2007. Clin. J. Sport Med..

[45] Krabak B.J., Parker K.M., DiGirolamo A. (2016). Exercise-Associated Collapse: Is Hyponatremia in Our Head?. PM R.

[46] Noakes T.D., Goodwin N., Rayner B.L., Branken T., Taylor R.K. (1985). Water Intoxication: A Possible Complication during Endurance Exercise. Med. Sci. Sports Exerc..

[47] Noakes T., IMMDA (2003). Fluid Replacement during Marathon Running. Clin. J. Sport Med..

[48] Montain S.J., Cheuvront S.N., Sawka M.N. (2006). Exercise Associated Hyponatraemia: Quantitative Analysis to Understand the Aetiology. Br. J. Sports Med..

[49] Shaltout H.A., Eggebeen J., Marsh A.P., Brubaker P.H., Laurienti P.J., Burdette J.H., Basu S., Morgan A., Dos Santos P.C., Norris J.L. (2017). Effects of Supervised Exercise and Dietary Nitrate in Older Adults with Controlled Hypertension and/or Heart Failure with Preserved Ejection Fraction. Nitric Oxide.

[50] Larsen F.J., Weitzberg E., Lundberg J.O., Ekblom B. (2007). Effects of Dietary Nitrate on Oxygen Cost during Exercise. Acta Physiol. Oxf..

[51] Domínguez R., Cuenca E., Maté-Muñoz J.L., García-Fernández P., Serra-Paya N., Estevan M.C.L., Herreros P.V., Garnacho-Castaño M.V. (2017). Effects of Beetroot Juice Supplementation on Cardiorespiratory Endurance in Athletes. A Systematic Review. Nutrients.

[52] McMahon N.F., Leveritt M.D., Pavey T.G. (2017). The Effect of Dietary Nitrate Supplementation on Endurance Exercise Performance in Healthy Adults: A Systematic Review and Meta-Analysis. Sports Med..

[53] Jonvik K.L., Nyakayiru J., van Loon L.J.C., Verdijk L.B. (2015). Can Elite Athletes Benefit from Dietary Nitrate Supplementation?. J. Appl. Physiol..

[54] Porcelli S., Pugliese L., Rejc E., Pavei G., Bonato M., Montorsi M., La Torre A., Rasica L., Marzorati M. (2016). Effects of a Short-Term High-Nitrate Diet on Exercise Performance. Nutrients.

[55] Clifford T., Constantinou C.M., Keane K.M., West D.J., Howatson G., Stevenson E.J. (2017). The Plasma Bioavailability of Nitrate and Betanin from Beta Vulgaris Rubra in Humans. Eur. J. Nutr..

[56] McIlvenna L.C., Monaghan C., Liddle L., Fernandez B.O., Feelisch M., Muggeridge D.J., Easton C. (2017). Beetroot Juice versus Chard Gel: A Pharmacokinetic and Pharmacodynamic Comparison of Nitrate Bioavailability. Nitric Oxide.

[57] Larsen F.J., Ekblom B., Sahlin K., Lundberg J.O., Weitzberg E. (2006). Effects of Dietary Nitrate on Blood Pressure in Healthy Volunteers. N. Engl. J. Med..

[58] Gomez-Cabrera M.-C., Borrás C., Pallardó F.V., Sastre J., Ji L.L., Viña J. (2005). Decreasing Xanthine Oxidase-Mediated Oxidative Stress Prevents Useful Cellular Adaptations to Exercise in Rats. J. Physiol..

[59] Gomez-Cabrera M.-C., Martínez A., Santangelo G., Pallardó F.V., Sastre J., Viña J. (2006). Oxidative Stress in Marathon Runners: Interest of Antioxidant Supplementation. Br. J. Nutr..

[60] Gomez-Cabrera M.-C., Domenech E., Romagnoli M., Arduini A., Borras C., Pallardo F.V., Sastre J., Viña J. (2008). Oral Administration of Vitamin C Decreases Muscle Mitochondrial Biogenesis and Hampers Training-Induced Adaptations in Endurance Performance. Am. J. Clin. Nutr..

[61] Gomez-Cabrera M.-C., Domenech E., Viña J. (2008). Moderate Exercise Is an Antioxidant: Upregulation of Antioxidant Genes by Training. Free Radic. Biol. Med..

[62] Vitale K.C., Hueglin S., Broad E. (2017). Tart Cherry Juice in Athletes: A Literature Review and Commentary. Curr. Sports Med. Rep..

[63] Bentley D.J., Ackerman J., Clifford T., Slattery K.S., Lamprecht M., Lamprecht M. (2015). Green Tea Catechins and Sport Performance. Antioxidants in Sport Nutrition.

[64] Rourke S. Drinking Tea: Are the Health Benefits Real?. http://www.medscape.com/viewarticle/907456.

[65] Kim J., Park J., Lim K. (2016). Nutrition Supplements to Stimulate Lipolysis: A Review in Relation to Endurance Exercise Capacity. J. Nutr. Sci. Vitaminol..

[66] Hursel R., Viechtbauer W., Dulloo A.G., Tremblay A., Tappy L., Rumpler W., Westerterp-Plantenga M.S. (2011). The Effects of Catechin Rich Teas and Caffeine on Energy Expenditure and Fat Oxidation: A Meta-Analysis. Obes. Rev..

[67] Palmatier M.A., Kang A.M., Kidd K.K. (1999). Global Variation in the Frequencies of Functionally Different Catechol-O-Methyltransferase Alleles. Biol. Psychiatry.

[68] Murase T., Haramizu S., Shimotoyodome A., Nagasawa A., Tokimitsu I. (2005). Green Tea Extract Improves Endurance Capacity and Increases Muscle Lipid Oxidation in Mice. Am. J. Physiol. Regul. Integr. Comp. Physiol..

[69] Murase T., Haramizu S., Shimotoyodome A., Tokimitsu I., Hase T. (2006). Green Tea Extract Improves Running Endurance in Mice by Stimulating Lipid Utilization during Exercise. Am. J. Physiol. Regul. Integr. Comp. Physiol..

[70] Partnerships Informed Choice. https://www.informed-choice.org/partnerships.

[71] Paluska S.A. (2003). Caffeine and Exercise. Curr. Sports Med. Rep..

[72] Goldstein E.R., Ziegenfuss T., Kalman D., Kreider R., Campbell B., Wilborn C., Taylor L., Willoughby D., Stout J., Graves B.S. (2010). International Society of Sports Nutrition Position Stand: Caffeine and Performance. J. Int. Soc. Sports Nutr..

[73] Glaister M., Gissane C. (2018). Caffeine and Physiological Responses to Submaximal Exercise: A Meta-Analysis. Int. J. Sports Physiol. Perform..

[74] Ivy J.L., Costill D.L., Fink W.J., Lower R.W. (1979). Influence of Caffeine and Carbohydrate Feedings on Endurance Performance. Med. Sci. Sports.

[75] Graham T.E., Hibbert E., Sathasivam P. (1998). Metabolic and Exercise Endurance Effects of Coffee and Caffeine Ingestion. J. Appl. Physiol..

[76] Graham T.E., Spriet L.L. (1995). Metabolic, Catecholamine, and Exercise Performance Responses to Various Doses of Caffeine. J. Appl. Physiol..

[77] Spriet L.L. (2014). Exercise and Sport Performance with Low Doses of Caffeine. Sports Med..

[78] Armstrong L.E., Casa D.J., Maresh C.M., Ganio M.S. (2007). Caffeine, Fluid-Electrolyte Balance, Temperature Regulation, and Exercise-Heat Tolerance. Exerc. Sport Sci. Rev..

[79] Lara B., Ruiz-Moreno C., Salinero J.J., Del Coso J. (2019). Time Course of Tolerance to the Performance Benefits of Caffeine. PLoS ONE.

[80] Beaumont R., Cordery P., Funnell M., Mears S., James L., Watson P. (2017). Chronic Ingestion of a Low Dose of Caffeine Induces Tolerance to the Performance Benefits of Caffeine. J. Sports Sci..

[81] Gonçalves L.S., Painelli V.S., Yamaguchi G., Oliveira L.F., Saunders B., da Silva R.P., Maciel E., Artioli G.G., Roschel H., Gualano B. (2017). Dispelling the Myth That Habitual Caffeine Consumption Influences the Performance Response to Acute Caffeine Supplementation. J. Appl. Physiol..

[82] Talanian J.L., Spriet L.L. (2016). Low and Moderate Doses of Caffeine Late in Exercise Improve Performance in Trained Cyclists. Appl. Physiol. Nutr. Metab..

[83] Nichols A.W. (2007). Probiotics and Athletic Performance: A Systematic Review. Curr. Sports Med. Rep..

[84] Spence L., Brown W.J., Pyne D.B., Nissen M.D., Sloots T.P., McCormack J.G., Locke A.S., Fricker P.A. (2007). Incidence, Etiology, and Symptomatology of Upper Respiratory Illness in Elite Athletes. Med. Sci. Sports Exerc..

[85] Leite G.S.F., Resende Master Student A.S., West N.P., Lancha A.H. (2019). Probiotics and Sports: A New Magic Bullet?. Nutrition.

[86] Coqueiro A.Y., de Oliveira Garcia A.B., Rogero M.M., Tirapegui J. (2017). Probiotic Supplementation in Sports and Physical Exercise: Does It Present Any Ergogenic Effect?. Nutr. Health.

[87] Pedersen D.J., Lessard S.J., Coffey V.G., Churchley E.G., Wootton A.M., Ng T., Watt M.J., Hawley J.A. (2008). High Rates of Muscle Glycogen Resynthesis after Exhaustive Exercise When Carbohydrate Is Coingested with Caffeine. J. Appl. Physiol..

[88] Davis W. (2014). Wheat Belly: Lose the Wheat, Lose the Weight, and Find. Your Path Back to Health.

[89] Perlmutter D. (2018). Grain Brain: The Surprising Truth about Wheat, Carbs, and Sugar—Your Brain’s Silent Killers.

[90] Piercy K.L., Troiano R.P., Ballard R.M., Carlson S.A., Fulton J.E., Galuska D.A., George S.M., Olson R.D. (2018). The Physical Activity Guidelines for Americans. JAMA.

[91] Duckworth A. (2016). Grit: The Power of Passion and Perseverance (Vol. 124).

[92] What You Need to Know about Dietary Supplements. https://www.fda.gov/food/dietarysupplements/usingdietarysupplements/ucm109760.htm.

